# In vitro activity of ferroquine (SSR 97193) against *Plasmodium falciparum *isolates from the Thai-Burmese border

**DOI:** 10.1186/1475-2875-6-81

**Published:** 2007-06-27

**Authors:** Marion Barends, Anchalee Jaidee, Nopparat Khaohirun, Pratap Singhasivanon, François Nosten

**Affiliations:** 1 Shoklo Malaria Research Unit, PO Box 46 Mae Sot, Tak 63110, Thailand; 2 Faculty of Tropical Medicine, Mahidol University, Bangkok, Thailand; 3 Centre for Vaccinology and Tropical Medicine, Churchill Hospital, Oxford, OX3 7LJ, UK

## Abstract

**Background:**

On the borders of Thailand, *Plasmodium falciparum *has become resistant to nearly all available drugs, and there is an urgent need to find new antimalarial drugs or drug combinations. Ferroquine (SSR97193) is a new 4-aminoquinoline antimalarial active against chloroquine resistant and sensitive *P. falciparum *strains *in vivo *and *in vitro*. This antimalarial organic iron complex (a ferrocenyl group has been associated with chloroquine) is meant to use the affinity of *Plasmodium *for iron to increase the probability for encountering the anti-malarial molecule.

The aim of the present study was to investigate the activity of ferroquine against *P. falciparum *isolates from an area with a known high multi-drug resistance rate.

**Methods:**

Parasite isolates were obtained from patients with acute falciparum malaria attending the clinics of SMRU. In vitro cultures of these isolates were set-up in the SMRU-laboratory on pre-dosed drug plates, and grown in culture for 42 hours. Parasite growth was assessed by the double-site enzyme-linked pLDH immunodetection (DELI) assay.

**Results:**

Sixty-five *P. falciparum *isolates were successfully grown in culture. The ferroquine mean IC_50 _(95% CI) was 9.3 nM (95% C.I.: 8.7 – 10.0). The mean IC50 value for the principal metabolite of ferroquin, SR97213A, was 37.0 nM (95% C.I.: 34.3 – 39.9), which is four times less active than ferroquine. The isolates in this study were highly multi-drug resistant but ferroquine was more active than chloroquine, quinine, mefloquine and piperaquine. Only artesunate was more active than ferroquine. Weak but significant correlations were found between ferroquine and its principal metabolite (r^2 ^= 0.4288), chloroquine (r^2 ^= 0.1107) and lumefantrine (r^2 ^= 0.2364).

**Conclusion:**

The results presented in this study demonstrate that the new ferroquine compound SSR97193 has high anti-malarial activity in vitro against multi-drug resistant *P. falciparum*.

## Background

The emergence of drug resistant malaria is a serious threat for malarial control [1,2]. Currently, chloroquine-resistant *Plasmodium falciparum *parasites are prevalent in most of the tropics, and in many areas resistance is high grade (i.e. potentially dangerous, with early treatment failures occurring) [1,2]. The rapid spread of chloroquine resistance has forced clinicians in many regions of the world to abandon classical therapy with chloroquine, in favour of other drugs that are less well tolerated and, importantly, more expensive. The situation in South-east Asia is of particular concern with increasingly frequent cases of 'multi-drug' resistant malaria [3]. On the borders of Thailand, *P. falciparum *has become resistant to nearly all available drugs [4,5]. These observations highlight the urgent need to find new antimalarial drugs or drug combinations and to develop optimal treatment protocols.

**Figure 1:**
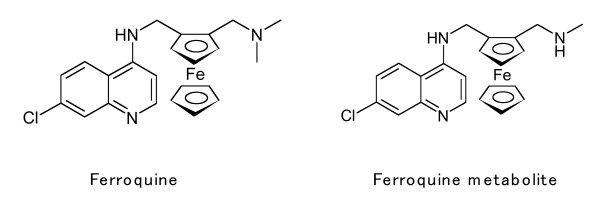
Chemical structure of ferroquine (7-chloro-4-[(2-N,N-dimethyl-aminomethyl) ferrocenylmethylamino]quinoline) and its principal metabolite.

Most new drugs arise from the identification of new therapeutic targets or metabolic pathways. Another approach is to modify an existing drug to enhance its activity [6]. Ferroquine (SSR97193) is a new 4-aminoquinoline antimalarial active against chloroquine resistant and sensitive *P falciparum *strains *in vivo *and *in vitro *[6-8] (Figure 1). This antimalarial organic iron complex (a ferrocenyl group has been associated with chloroquine) is meant to use the affinity of *Plasmodium *for iron to increase the probability for encountering the anti-malarial molecule [6-9]. The ferrocene group by itself does not have antimalarial activity [8].

Four previous studies had investigated the activity of ferroquine against *P. falciparum *isolates from infected patients [10-13] (Table 1). However, the drug sensitivity of *P. falciparum *strains varies between different locations, where *P. falciparum *isolates have different anti-malarial resistance backgrounds.

**Table 1:** Publications on the activity of ferroquine against *P. falciparum *isolates

					***Drug IC50 (Geometric mean)***		
						
	***Year***	***Country***	***Region***	***No. of isolates***	***Ferroquine***	***Chloroquine***	***Method***
Pradine *et al*. [10]	2001	Gabon	Libreville	103	10.8 nM	370 nM	Isotopic microtest
Atteke *et al*. [12]	2003	Gabon	Franceville	56	16.0 nM	141.3 nM	Isotopic microtest
			Bakoumba	60	27.9 nM	398.0 nM	
Pradine *et al*. [11]	2002	Senegal		55	7.9 nM	102 mM	Isotopic microtest
Chim *et al*. [13]	2004	Cambodia		127	30.61 nM	125.04 nM	Isotopic microtest

The aim of the present study was to confirm the efficiency of ferroquine against *P. falciparum *isolates from patients treated for malaria in one of the SMRU clinics along the Thai-Burmese border. This area has a known high multi-drug-resistance rate, with very high resistance against chloroquine [14].

## Methods

### Isolates of *Plasmodium falciparum*

Between November 2004 and May 2005 parasite isolates were obtained from patients with acute falciparum malaria attending the clinics of the Shoklo Malaria Research Unit (SMRU). The clinics are open for both migrants and refugees and are situated in an area of forested hills on the northwestern border of Thailand. Isolates were collected if the parasitaemia was at least 0.1%, by taking 5 cc whole blood by venopunture into sterile Vacutainer^® ^tubes containing 0.05 ml K3 EDTA. The blood samples were transported within 4–6 hours at room temperature and then set up in continuous culture upon their arrival in the SMRU laboratory in Mae Sot, not more than one-hour drive from the study sites. Samples were only provided by patients who gave written, informed consent following written and oral explanations given in their own language. This study was part of a series of treatment trials approved by the Ethical Committee of the Faculty of Tropical Medicine, Mahidol University, Bangkok, Thailand.

### In vitro drug assay

Ferroquine (FQ) (SSR97193: 7-chloro-4-[(2-N,N-dimethyl-aminomethyl) ferrocenylmethylamino]quinoline), and ferroquine metabolite (FQM) (SR97213A: C_22_H_22_ClFeN_3_) (Figure 1) were obtained from Sanofi-Synthelabo-Recherche, France. Chloroquine diphosphate (CQ), quinine citrate (QN) and doxycyline hydrochloride (DOX) were obtained from Sigma Chemicals, UK. Lumefantrine (LUM) was obtained from Novartis Pharmacia, Basel, Switzerland. Sodium artesunate (AS), piperaquine (PIP) and mefloquine (MFQ) were kindly donated by Dr. Niklas Lindegård, Faculty of Tropical Medicine, Mahidol University, Thailand. FQ and FQM were dissolved in DMSO, QN, MFQ and AS in 70% ethanol, CQ and DOX in deionised water, PIP in 100% methanol and LUM was dissolved in a 1:1:1 (w/v) mixture of ethanol, Triton-X (Sigma), linoleic acid (Sigma). All drugs were dissolved initially at a concentration of 1 mg/ml, and serial dilutions were made in complete RPMI medium. The solvent in the final concentrations had no significant effect on parasite growth when compared to culture media. All concentrations, including drug-free controls, were distributed in duplicate in 96-well tissue culture plates. The drug-plates were made in bulk and stored at -80°C until use (for up to three months).

For each sample, plasma and buffy coat were removed after centrifugation and the red cells washed three times in phosphate buffered saline (PBS). The infected red blood cells were set-up in the pre-dosed drug plates in complete RPMI with 10% heterologous sera, at a parasitaemia of 0.1% parasitized erythrocytes and a haematocrit of 1.5%. The plates were incubated at 37°C in the presence of 5% CO_2_, 90% N_2 _and 5% O_2 _for 42 hours. After culture the plates were frozen down at -20°C.

The chloroquine-resistant *P. falciparum *laboratory clone K1 was used for quality control of the drug-plates.

### DELI

The double-site enzyme-linked pLDH immunodetection (DELI) assay was used to assess *P. falciparum *antimalarial drug susceptibility. The DELI method was performed as described previously [15,16]. In brief, the culture plates were thawed and frozen three times order to lyse the cells. 100 μl from each well were transferred into 96-well plates (Nunc-Immuno™ plate, maxisorb, Nalgene Nunc International, Denmark) pre-coated with a capture monoclonal antibody 17E4, which specifically recognizes the pLDH, incubated for 1 hour at 37°C. Following washing 3× with PBS/0.5% bovine serum albumin (BSA Fraction V) (Roche Diagnostics, Mannheim, Germany), a second biotinylated anti-pLDH monoclonal antibody 19G7 was added and the plates incubated for 1 hour at 37°C. After removal of unbound antibody by washing 3× with PBS/0.5% BSA, the plates were incubated at room temperature for 30 minutes with a 1:10,000 solution of streptavidin-POD conjugate (Roche Diagnostics). After washing the plates 3× with PBS/0.5% BSA, the plates were incubated for up to 20 minutes at room temperature with a peroxidase substrate solution, 3,3',5,5'-tetramethylbenzidine (KPL, Maryland, USA). The reaction was stopped with 1 M phosphoric acid and colour development was quantified immediately using a spectrophotometer to determine the OD at 450 nm with a reference filter at 690 nm.

### Analysis of dose response curves

Dose response curves, IC_50 _values, and coefficients of variation were calculated by fitting the data to an inhibitory E-max pharmacokinetic model using WINNONLIN Ver 4.1 (Pharsight Corporation). To ensure data quality we rejected all IC_50 _values with coefficients of variation (Standard Error × 100)/Mean) of estimated IC_50 _values > 30% and those in which the pLDH production in control wells (parasites, no drug) was < 5 times background (red blood cells only).

### Statistical analysis

Data were analysed using the program SPSS 11.0 for Windows (SPSS Inc., Chicago, Illinois, USA). Prior to analysis, *in vitro *drug response data were normalized by log transformation. Ferroquine cross-resistance with the other antimalarials (metabolite, chloroquine, artesunate, quinine, mefloquine, lumefantrine, doxycyclin and piperaquine) was estimated by Pearson correlation coefficient (*r*) and coefficient of determination (*r*^2^).

## Results

Sixty-five *P. falciparum *isolates from non-pregnant patients were successfully grown in culture and gave interpretable results for the calculation of the mean drug IC50 values as shown in Table 2. The ferroquine mean IC_50 _was 9.30 (95% C.I.: 8.69 – 9.96) nM, with individual values ranging from 3.86 to 18.23 nM. The mean IC50 value for ferroquine metabolite, the principal metabolite of ferroquine, was 37.00 nM (95% C.I.: 34.32 – 39.89), which is almost 4 times less active than ferroquine. All isolates were resistant against chloroquine using the standard cut-off IC50 of 100 nM. The mean IC50 value for chloroquine was 340.75 nM (95% C.I.: 304.04 – 381.89), which makes ferroquine 36-fold more potent than chloroquine in these isolates. A weak but significant correlation was found between ferroquine with ferroquine metabolite (*r *= 0.655, *r*^2 ^= 0.4288, *P *= 0.0001) and ferroquine with chloroquine (*r *= 0.333, *r*^2 ^= 0.1107, *P *= 0.009 (Table 2). Also a significant correlation was observed between the IC50 for ferroquine and quinine. However this correlation was not reproducible in a second set of samples. No significant correlation between ferroquine and artesunate was detected.

**Table 2:** The *in vitro *IC50 responses of the 65 isolates of *Plasmodium falciparum *to ferroquine, ferroquine metabolite, chloroquine, artesunate and quinine. Ferroquine cross-resistance with the other antimalarials was estimated by Pearson correlation coefficient (*r*), and coefficient of determination (*r*^2^)

		**Mean inhibitory concentration (nM)**				
					
**Drug**	No. of isolates	**Geometric mean**	**95% confidence interval**	***r***	***r*^2^**	P
Ferroquine	65	9.30	8.69 – 9.96	-	-	-
FQ-Metabolite	64	37.00	34.32 – 39.89	0.655	0.4288	0.0001
Chloroquine	62	340.75	304.04 – 381.89	0.333	0.1107	0.0090
Artesunate	56	4.02	3.06 – 6.28	-0.169	0.0284	0.2190
Quinine	49	1016.05	894.36 – 1154.29	0.338	0.1145	0.0200

For a subset of the *P. falciparum *isolates (n = 22) the IC50 values for doxycycline, lumefantrine, mefloquine and piperaquine were analysed as well (Table 3). These data showed that ferroquine was more active than any of these drugs in this group of isolates. Only for lumefantrine a significant correlation with ferroquine was found (r = 0.486, *r*^2 ^= 0.2364, *P *= 0.025).

**Table 3:** The *in vitro *IC50 responses of a subset of *Plasmodium falciparum *isolates (n = 22) to Ferroquine, Ferroquine metabolite, Doxycyxline, Lumefantrine, Mefloquine, and Piperaquine. Ferroquine cross-resistance with the other antimalarials was estimated by Pearson correlation coefficient (*r*), and coefficient of determination (*r*^2^).

		**Mean inhibitory concentration (nM)**				
					
**Drug**	**No. of isolates**	**Geometric mean**	**95% confidence interval**	***r***	***r*^2^**	***P***
Ferroquine	21	8.91	8.17 – 9.72	-	-	-
FQ-Metabolite	22	31.40	28.16 – 35.01	0.674	0.4542	0.001
Doxycycline	20	2719.85	2236.38 – 3307.09	-0.203	0.0411	0.391
Lumefantrine	22	11.77	10.31 – 13.44	0.486	0.2364	0.025
Mefloquine	21	144.23	125.82 – 165.33	0.296	0.0874	0.206
Piperaquine	19	49.20	37.16 – 65.11	0.017	0.0003	0.946

## Discussion

This study confirms that ferroquine (SSR97193) is highly active against *P. falciparum in vitro*. The *P. falciparum *isolates analysed in this study were highly multi-drug resistant but ferroquine was more active than chloroquine, quinine, mefloquine, piperaquine and doxycycline. Only artesunate was more active than ferroquine. In addition, ferroquine was four times more active *in vitro *than the tested principal ferroquine metabolite (SR97213A). This underlines the importance to test the biological activity of the plasma and whole blood of treated patients or volunteers, as other metabolites may be active.

Weak but significant correlations between the response to ferroquine and that to chloroquine and lumefantrine were found. The high activity of ferroquine on chloroquine-resistant *P. falciparum *(the lowest CQ IC50 analysed is 102.4 nM) suggests either that both drugs have different modes of action, or that ferroquine reverses chloroquine resistance [12]. Chloroquine is believed to act by concentrating in the parasite digestive vacuole and preventing the conversion of toxic heme to haemozoin, leading to membrane damage and parasite death [17,18]. Biot *et. al*. recently demonstrated that ferroquine, like chloroquine, forms complexes with haematin and is an even stronger inhibitor of β-haematin formation than chloroquine [9]. Chloroquine-resistant parasites expel chloroquine much more rapidly from red blood cells than chloroquine-sensitive parasites. This efflux of chloroquine is catalyzed by a *P. falciparum *transmembrane protein (*Pf*CRT) [18]. Ferroquine may block the *Pf*CRT through its lipophilic properties, acting like a resistance-reversing agent [9].

So far, no resistance of *P. falciparum *to ferroquine has been found *in vitro *either in cultures of patient isolates or in laboratory-adapted strains under drug pressure [18].

In Table 1, the published studies on the *in vitro *susceptibility to ferroquine and chloroquine are summarized. The isolates tested in the present study show comparable *in vitro *sensitivity to ferroquine to that found in Gabon (Libreville) [10] and Senegal [11]. Isolates from Cambodia have the highest IC50 values for ferroquine. However, caution must be exerted when comparing these results because in the present study a DELI method rather than an isotopic assay is used. Previous reports have demonstrated that DELI does slightly overestimate the IC50 for chloroquine and lumefantrine, and underestimate for artesunate, compared to the isotopic microtest [14]. No data on the direct comparison of DELI with the isotopic microtest for ferroquine levels are available as yet.

The results presented in this study indicate that ferroquine is active *in vitro *regardless of high grade multi-drug resistance. Still further research is needed to elucidate the mode of action of ferroquine and identify the putative molecular markers of resistance. In addition, since chloroquine resistance is also found in *Plasmodium vivax *[19], the activity of ferroquine to *P. vivax *should be studied.

## Competing interests

The author(s) declare that they have no competing interests.

## Authors' contributions

MB carried out the study and analysed the data. AJ and NK carried out the parasite cultures and ELISA assays. MB, PS and FN participated in the study design and contributed to draft the manuscript. All authors read and approved the final manuscript.
